# Mesh-Augmented Ventral Hernia Repair Despite Iatrogenic *Staphylococcus aureus*-Peritonitis Due to Progressive Pneumoperitoneum: A Case Report

**DOI:** 10.70352/scrj.cr.25-0099

**Published:** 2025-07-11

**Authors:** Robin Klewitz, Magdalena Menzel, Philipp Holzner, Stefan Fichtner-Feigl, Julian Hipp

**Affiliations:** Department of General and Visceral Surgery, Medical Center, University of Freiburg, Freiburg, Germany

**Keywords:** ventral hernia repair, loss of domain, progressive pneumoperitoneum, perioperative complications

## Abstract

**INTRODUCTION:**

Loss-of-domain in large incisional hernia needs to be addressed by mesh-augmented repair combined with a combination of component separation techniques: progressive pneumoperitoneum (PPP) and chemical component separation with botulinum toxin A. In this case report, successful management of an iatrogenic *Staphylococcus aureus* peritonitis caused by PPP with nevertheless definitive treatment of a giant loss-of-domain ventral hernia is presented.

**CASE PRESENTATION:**

A female patient with M1-3W3 recurrent incisional hernia with a loss-of-domain of 47% was prepared for definitive ventral hernia repair by chemical component separation with botulinum toxin A-infiltration and PPP via an intraperitoneally placed central venous catheter. A significant increase of inflammatory markers was found after 28 days. An emergency CT scan was performed, which showed the PPP and perihepatic/perisplenic contrast-enhancing fluid collections. Exploratory laparoscopy and laparotomy revealed no bowel perforation but fibrinous peritonitis due to an iatrogenic PPP-catheter-associated peritonitis. Despite the fibrinous peritonitis, we decided to proceed with definitive ventral hernia repair (Rives-Stoppa-Sublay-Herniotomy with transversus abdominis release (left) and anterior component separation (right), 42 × 30 cm permanent polypropylene mesh). Initial calculated antibiotic treatment was performed with piperacillin/tazobactam. Microbiologic examinations revealed *Staphylococcus* *aureus* in the intraoperative specimens on postoperative day 1 and the antibiotic treatment was changed to intravenous flucloxacillin for 14 days after surgery. The further hospital stay was uneventful and the patient was discharged on the 20th postoperative day.

**CONCLUSIONS:**

The presented case demonstrates the possibilities in complex ventral hernia repair to achieve a satisfying outcome for the patients. Even in cases with infectious complications, a single-stage procedure might be performed safely and a complete reconstruction of the abdominal wall might be achieved. The risk of chronic mesh infection in contaminated situations, especially during the presence of *Staphylococcus aureus*, remains uncertain and has to be weighed against possible benefits.

## INTRODUCTION

The development of an incisional hernia is a common complication of abdominal surgery. Large incisional hernia can present as loss-of-domain (LOD) hernia, if the hernia sac volume represents more than 20% of the total peritoneal volume. In these cases, fascial closure is not possible without additional technical measures. Component separation techniques, progressive pneumoperitoneum (PPP), chemical component separation with the injection of botulinum toxin A, or a combination of the aforementioned procedures are options to achieve fascial closure.^1)^ Although peritonitis is a rarely reported complication of PPP, the risk of mesh infection is ever present in mesh augmented repairs of ventral incisional hernia and the use of permanent mesh in contaminated fields is discussed controversially.^2)^ In this case report, we want to share our experience with the successful management of an iatrogenic *Staphylococcus aureus*-peritonitis caused by PPP with nevertheless definitive treatment of a giant LOD ventral hernia.

## CASE PRESENTATION

We present the case of a 60-year-old female patient with a history of prior vertical gastroplasty 20 years ago due to adiposity (initial/maximal body mass index (BMI): 60.1 kg/m^2^). In the following years, several revisional surgical procedures were performed, including 2 attempts of direct suture repair of an incisional hernia of the median laparotomy.

The first presentation of the patient in our department was in the center for bariatric surgery 2 years ago. The patient had a symptomatic large recurrent incisional hernia and a BMI of 52.1 kg/m^2^. A multimodal concept consisting of initial bariatric surgery for optimal weight loss and control of comorbidities, followed by definitive repair of the recurrent hernia was planned. The patient underwent an initial medical treatment of the morbid adiposity with Semaglutid, followed by open revisional bariatric surgery and a one-anastomosis gastric bypass was established. Significant weight loss was achieved (BMI: 39.7 kg/m^2^) and the patient was transferred to the abdominal wall reconstruction team of our department to initiate preparation for definitive surgery of the incisional hernia.

**Fig. 1:**
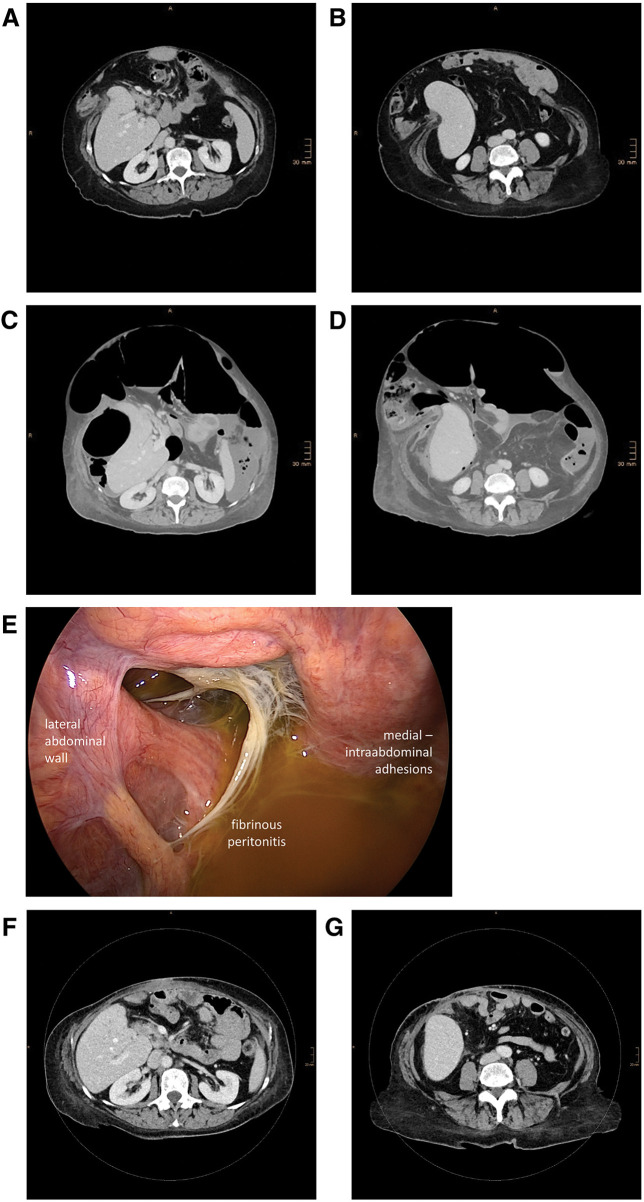
Preinterventional, preoperative, intraoperative, and postoperative aspects of the complex recurrent incisional hernia. (**A**, **C**, and **F** are on the same level (lumbar vertebra L2–hilum of kidneys) and **B**, **D**, and **G** are on the same level (lumbar vertebra L4–caudal edge of kidneys)). Preinterventional: (**A** and **B**) Preinterventional CT scan of the recurrent incisional hernia with 47% loss-of-domain prior to the chemical component separation and establishment of the PPP. Preoperative: (**C** and **D**) CT scan performed preoperatively due to increased inflammatory markers. The CT scan shows the iatrogenic PPP and perihepatic and perisplenic fluid collections. Intraoperative: (**E**) Laparoscopic view of the right upper quadrant. Fibrinous peritonitis with ascites can be detected. Postoperative: (**F** and **G**) 7-month postoperative CT scan. Complete restoration of the abdominal wall integrity after Rives-Stoppa-Sublay-Herniotomy with bilateral component separation is shown in the scan. No signs of recurrence or fluid collections/mesh infection are present.

Clinical re-examination and CT (**Fig. 1A** and **1B**) showed a recurrent M1-3W3 incisional hernia of 20 × 18 cm with an LOD of 47% calculated by Sabbagh’s method. In this method, the LOD is calculated as the percentage of the total peritoneal volume (TPV) (LOD = hernia sac volume (HSV)/(HSV + abdominal cavity volume (ACV)) or LOD = HSV/TPV. For the presented case: ACV: 3593 mL; HSV: 3187 mL; LOD = 47%).^3)^ The calculations were performed based on CT-volumetry done with the software syngo.via (Siemens Healthineers, Forchheim, Germany). The preparation for definitive ventral hernia repair was started by chemical component separation with botulinum toxin A-infiltration (3 infiltration-sites per side, injection of botulinum toxin A in each muscle layer at each site (total of 18 injections) under ultrasonographic visualization; in total 300 IE botulinum toxin A in 150 mL NaCl 0.9%) of the lateral abdominal wall muscles and subsequent establishment of a PPP via an intraperitoneally placed central venous catheter as previously reported.^1,4)^ A standard 1-lumen central venous catheter (14 Ga./2.2 mm, 20 cm length, Arrowg+ard Blue; Teleflex, Morrisville, NC, USA) was placed in the right upper quadrant under ultrasonographic visualization in the peritoneal cavity. A first bolus of 1000 mL ambient air was insufflated and after that, a CT scan confirmed intraabdominal location of the catheter and the pneumoperitoneum. The target volume of the PPP was calculated according to Bueno-Lledó et al.;^1)^ therefore, the target volume was 3 × HSV (3187 mL) ≈ 9–10 L of ambient air. No target pressure was defined and no pressure monitoring during PPP was performed.

Over a period of 7 out-patient visits (23 days), a progressive pneumoperitoneum was established with a total intraperitoneal air volume of 10.6 L with a PPP maintenance period of 7 days after the last insufflation session. Surgical treatment was planned for day 29. On day 28, the routine preoperative blood sample showed a significant increase of inflammatory markers with a C-reactive protein of 376 mg/L and a white blood cell count of 15.4 × 10^3^/µL. Due to this finding, an emergency CT scan was performed, which showed the iatrogenic PPP but also ascites and perihepatic and perisplenic contrast-enhancing fluid collections (**Fig. 1C** and **1D**). An empiric antibiotic therapy was initiated with piperacillin/tazobactam prior to emergency surgery.

We performed an urgent laparoscopy (**Fig. 1E**), but a conversion to laparotomy was deemed necessary, as a complete examination of the abdominal cavity was not possible due to severe intraabdominal adhesions. Eventually, we found fibrinous peritonitis in the upper abdomen without a sign of bowel/gastric perforation and an iatrogenic PPP-catheter-associated peritonitis was suspected. Despite the fibrinous peritonitis (Class IV (dirty-contaminated) according to CDC-classification^5)^), we decided to proceed with definitive ventral hernia repair, as the definitive closure of the abdominal wall with restoration of the linea alba would not be possible once the PPP was desufflated.

After adhesiolysis and lavage of the abdominal cavity with sterile Ringer solution, we performed a Rives-Stoppa-Sublay-Herniotomy with posterior component separation (transversus-abdominis-release, TAR) on the left side and anterior component separation (Ramirez-Plasty) on the right side. At first, anterior component separation was performed on the right side, as the persisting gap between both sides of the linea alba was still very large after preparation of both retrorectus spaces. The right side was chosen for the anterior component separation, as the retrorectus space was wider after the initial Rives-Stoppa-preparation on this side. After one-sided anterior component separation, the remaining gap in the linea alba decreased significantly, so that a posterior component separation was deemed sufficient for successful reconstruction of the linea alba. With the one-sided TAR, the mesh overlap can be optimized on this side and the risk for postoperative subcutaneous seroma is reduced. We therefore choose TAR whenever clinically appropriate over anterior component separation in our approach to abdominal wall reconstruction.

A 42 × 30 cm polypropylene mesh (Prolene Soft Mesh; Ethicon, Somerville, NJ, USA) was placed in a diamond configuration in the retrorectus space and was fixated by transfascial sutures. Fascial closure was facilitated with intraoperative fascial traction for 30 min with subsequent primary reconstruction of the linea alba.^6)^ Finally, wound closure was done with a prophylactic vacuum assisted closure-system (V.A.C.).

After surgery, the patient was treated in our surgical intensive care unit. Without the microbiologic results yet available, initial calculated antibiotic treatment was performed with piperacillin/tazobactam. On the 1st postoperative day, *Staphylococcus* *aureus* (methicillin-sensitive *Staphylococcus aureus*, MSSA) was identified in several intraoperative specimens and the antibiotic treatment was changed to intravenous flucloxacillin (4 × 2 g/day) for 14 days after surgery. The further hospital stay was uneventful, the wound was secondarily closed on the 14th postoperative day after 2 changes of the V.A.C. system (5th and 10th postoperative day), and the patient was discharged on the 20th postoperative day.

In the 7-month follow-up-examination, the patient was doing well without any signs of chronic mesh infection or recurrence (**Fig. 1F** and **1G**).

## DISCUSSION

To our knowledge, this is the first reported case of a *Staphylococcus* *aureus*-peritonitis caused by PPP with nevertheless successful definitive treatment of a giant LOD ventral hernia. Peritonitis is a rarely reported complication of PPP,^7)^ but *Staphylococcus aureus* infections of synthetic mesh are generally considered difficult to manage as bacterial biofilms can form on synthetic implants. In many cases, staged procedures with primary control of the septic focus and secondary restoration of the abdominal wall anatomy with definitive hernia repair and mesh implantation after 6–12 months is commonly used, although evidence for the use of synthetic non-absorbable mesh in contaminated fields is increasing.^2,8–11)^ Prior low-level evidence with a limited number of cases exists for the possibility of synthetic mesh implantation despite *Staphylococcus aureus* infection.^11,12)^ It is worth noting that the choice of implant may play a significant role in the management of such cases. In this case, we used a medium-weight non-absorbable large-pore polypropylene mesh (Prolene Soft Mesh; Ethicon). Especially polypropylene mesh was able to clear a large percentage of *Staphylococcus* *aureus* contamination in a prior *in vivo* study, while composite meshes are prone to persisting foreign body infection.^13)^ Therefore, the polypropylene mesh used in our case seems to be a valid choice, however, prospective clinical evidence regarding the choice of mesh material is missing. Besides the mesh material used, the early start of an effective antibiotic treatment, extensive intraoperative lavage, and the use of a prophylactic V.A.C. therapy may be factors influencing the positive patient outcome in this clinical scenario.

Other possible complications occurring during PPP have been studied by a recent systematic review.^7)^ The overall complication rate of PPP is 12.4% and the overall mortality rate of PPP is 0.4%. The rate of intraabdominal infection due to PPP is reported to be 0.6% in this review. The main complications of PPP are retroperitoneal/mediastinal emphysema, shoulder pain, abdominal pain, respiratory insufficiency/dyspnea, and intraabdominal infections.^7)^ Shoulder pain and abdominal pain should be treated with a slower establishment of the PPP, so that the patient can adapt to the abdominal distension. In the case of emphysema, a CT scan is mandatory to evaluate the correct placement of the catheter used for PPP insufflation. Depending on the patient’s condition, continuation of the PPP has to be evaluated critically in cases with emphysema or dyspnea/respiratory insufficiency. Intraabdominal infections have to be treated according to the site of infection, the timing of onset during PPP, and other factors. Whether a successful treatment of the patient’s hernia is possible with concomitant infectious complications has to be evaluated for every single case. Our case report shows, however, that definitive mesh-augmented treatment is an option for these patients, if the possible benefits outweigh the risks involved to this approach.

## CONCLUSIONS

In conclusion, a 2-staged procedure would not have been possible in this clinical scenario without sacrificing the efforts of the established PPP, as the reconstruction of the abdominal wall would not have been possible without a new PPP establishment in the future. The determined pursuance of the aim to reconstruct the patient’s abdominal wall was necessary to achieve this goal. The presented case demonstrates the possibilities in complex ventral hernia repair to achieve a satisfying outcome for the patients. Even in cases with infectious complications, a single-stage procedure might be performed safely and a complete reconstruction of the abdominal wall might be achieved. Nevertheless, the risk of chronic mesh infection in contaminated situations, especially during the presence of *Staphylococcus* *aureus*, remains uncertain and has to be weighed against possible benefits in the individual clinical situation of the patient.

## DECLARATIONS

### Funding

No funding was received.

### Authors’ contributions

Conceptualization: R.K. and J.H.

Investigation: R.K., M.M., P.H., and J.H.

Writing - original draft: R.K. and J.H.

Writing - review & editing: R.K., M.M., P.H., S.F.F., and J.H.

All authors have read and approved the manuscript.

The authors are responsible for the manuscript.

### Availability of data and materials

Further details regarding the presented case are available from the corresponding author upon reasonable request.

### Ethics approval and consent to participate

Ethics approval was not necessary for this case report. Written consent to participate is obtained from the patient.

### Consent for publication

Written informed consent for publication of this case report is obtained from the patient.

### Competing interests

The authors declare no conflict of interest.
